# Biological Role and Aberrant Overexpression of Syntenin-1 in Cancer: Potential Role as a Biomarker and Therapeutic Target

**DOI:** 10.3390/biomedicines11041034

**Published:** 2023-03-27

**Authors:** Valeria Guadalupe Pintor-Romero, Edgar Hurtado-Ortega, María Lilia Nicolás-Morales, Mayralina Gutiérrez-Torres, Amalia Vences-Velázquez, Carlos Ortuño-Pineda, Mónica Espinoza-Rojo, Napoleón Navarro-Tito, Karen Cortés-Sarabia

**Affiliations:** 1 Laboratorio de Inmunobiología y Diagnóstico Molecular, Facultad de Ciencias Químico Biológicas, Universidad Autónoma de Guerrero, Chilpancingo de los Bravo 39086, Mexico; 2 Laboratorio de Ácidos Nucleicos y Proteínas, Facultad de Ciencias Químico Biológicas, Universidad Autónoma de Guerrero, Chilpancingo de los Bravo 39086, Mexico; 3 Laboratorio de Biología Molecular y Genómica, Facultad de Ciencias Químico Biológicas, Universidad Autónoma de Guerrero, Chilpancingo de los Bravo 39086, Mexico; 4 Laboratorio de Biología Celular del Cáncer, Universidad Autónoma de Guerrero, Chilpancingo de los Bravo 39086, Mexico

**Keywords:** syntenin-1, cancer, therapeutic target, biomarker

## Abstract

Syntenin-1 is a 298 amino acid protein codified by the melanoma differentiation-associated gene-9 (*MDA-9*). Structurally, it is composed of four domains: N-terminal, PDZ1, PDZ2, and C-terminal. The PDZ domains of syntenin-1 are involved in the stability and interaction with other molecules such as proteins, glycoproteins, and lipids. Domains are also associated with several biological functions such as the activation of signaling pathways related to cell-to-cell adhesion, signaling translation, and the traffic of intracellular lipids, among others. The overexpression of syntenin-1 has been reported in glioblastoma, colorectal, melanoma, lung, prostate, and breast cancer, which promotes tumorigenesis by regulating cell migration, invasion, proliferation, angiogenesis, apoptosis, and immune response evasion, and metastasis. The overexpression of syntenin-1 in samples has been associated with worst prognostic and recurrence, whereas the use of inhibitors such as shRNA, siRNA, and PDZli showed a diminution of the tumor size and reduction in metastasis and invasion. Syntenin-1 has been suggested as a potential biomarker and therapeutic target in cancer for developing more effective diagnostic/prognostic tests or passive/active immunotherapies.

## 1. Introduction

The melanoma differentiation-associated gene-9 (*MDA-9*), also known as syntenin-1 or syndecan binding protein (SDCBP), belongs to the PDZ family, and its name derives from the proteins in which this domain was first described: PSD-95 (postsynaptic density protein-95), DLG (drosophila disc large tumor suppressor protein), and ZO-1 (zonula occludens-1). Syntenin-1 was initially identified as an interactive partner of syndecans and for being involved in cell surface signaling and protein trafficking [1,2,3]. At the same time, this protein was identified as a gene product derived from the differentiation of human melanoma cells treated with interferon β (IFN-β) and Mezerein (MEZ) [2]. Structurally, syntenin-1 is composed of four domains: two PDZ domains (PDZ1 and PDZ2), one N-terminal domain (NTD), and one C-terminal domain (CTD) [4]. PDZ domains are responsible for the functional part and recognize the CTD of target proteins [5]. It has been described that most of the organs in adults express basal levels of syntenin-1 [2,6] and contributes to the physiological process as cell-to-cell adhesion, traffic, cellular signaling, transcription regulation, immunological synapse, axonal growth regulation, architecture, and neuronal synapsis, among others. However, recent studies have shown that syntenin-1 expression is higher in neurons and tumor cells compared to other cell types [7]. Particularly in cancer, the overexpression of syntenin-1 has been reported in breast [8], prostate [9], lung [10], and colorectal [11] cancer, as well as glioblastoma [12]. In cancer, syntenin-1 participates in the metastatic process, including cell migration and invasion, angiogenesis, and epithelial-mesenchymal transition (EMT) of tumor cells [13]. Given the significant potential role of syntenin-1 during cancer development, it has been proposed as a potential therapeutic target. Interestingly, inhibitors targeting specific sites of syntenin-1 have been developed, and a diminution in the invasiveness and migration of tumor cells has been observed [11,14,15].

## 2. Syntenin-1

Syntenin-1 was described for the first time during the search for new interaction partners of syndecans. One year later, Lin et al. reported the *MDA-9* gene in human melanoma cells treated with IFN-β and the antileukemic agent MEZ. This gene is located in chromosome 8q12 (from 58,553,169 bp to 58,582,860 bp) and has 9 exons; the cDNA has a length of 2.1 kb and an open reading frame of 894 bp that encodes for a polypeptide chain of 298 amino acids (aa) with a molecular weight of 33 kDa. Syntenin-1 is an adapter or scaffolding protein and belongs to the PDZ family [1,2]. PDZ domains are located in more than 400 copies of the human genome, including multidomain cytoplasmatic proteins associated with the orientation to receptors and channels in the cell membrane and the assembly of the supramolecular signaling complex [16]. Structurally, they are represented as preserved modules with a length of 90 amino acids and 8 segments (6 β-sheets and 2 α-helix), which fold into a 6-stranded β sandwich [5,17].

**Figure 1:**
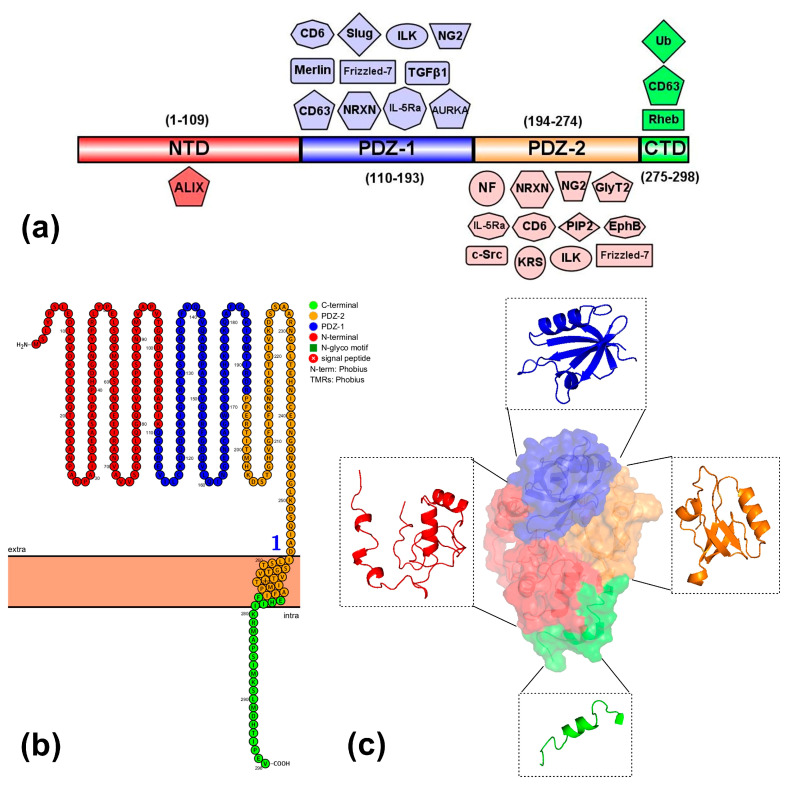
Structure and domains of syntenin-1. (**a**) The linear structure of syntenin-1 (access number NCBI: NP_001007068.1) shows the four structural domains and their localization (aa): N-terminal domain (NTD), PDZ1 and PDZ2 domains, and C-terminal domain (CTD). Protein and multiprotein complexes associated with each domain are also illustrated. NTD: ALIX (ALG-2-interacting protein X); PDZ1: NRXN (Neurexins), Merlin, CD6 (Cluster of differentiation 6), IL-5Rα (Interleukin-5 Receptor alpha), CD63, NG2 (Neural/glial antigen 2), Fzd7 (Frizzled 7), ILK (Integrin-linked kinase), Slug, TGFβ1 (Transforming growth factor beta 1), and AURKA (Aurora Kinase A); PDZ-2: NF (Neurofascin), NRXN (Neurexins), Syndecan, GlyT2 (Glycine transporter 2), IL-5Rα (Interleukin-5 Receptor alpha), c-Src (Proto-oncogene tyrosine-protein kinase Src), KRS (Lysyl-tRNA Synthetase), PIP2 (Phosphatidylinositol 4,5-bisphosphate), NG2 (Neural/glial antigen 2), Fzd7 (Frizzled 7), EphB (Ephrin B), and ILK (Integrin-linked kinase); CTD: Rheb (Ras homolog enriched in brain), CD63, and Ub (Ubiquitin). Created in IBS (Illustrator for Biological Sequences: http://ibs.biocuckoo.org/, accessed on 4 February 2023). Schematic representation of the 2D (https://wlab.ethz.ch/protter/, accessed on 5 February 2023) (**b**) and 3D structure (**c**) of syntenin-1. In red: NTD (1–109 aa); blue: PDZ1 (110–193 aa); orange: PDZ2 (194–274 aa); and green: CTD (275–298 aa). In the 2D structure (**b**), the extracellular, transmembrane, and cytoplasmic domains are shown, whereas, in the 3D structure (**c**), the surface and alpha helix/β-sheets are illustrated. The 3D structure was obtained by ab initio structure modeling in the server RaptorX (http://raptorx6.uchicago.edu/, accessed on 5 August 2022).

MDA-9/Syntenin-1 contains 4 structural domains: NTD (1–109 aa), PDZ-1 domain (110–193 aa), PDZ-2 domains (194–274 aa), and a short CTD (275–298 aa) [4]. PDZ domains are joined by a short linker (Arg^193^-Pro^194^-Phe^195^-Glu^196^) and, similarly to other members of the PDZ superfamily, show a typical fold with two opposite antiparallel β-sheets capped by two α-helix. Each domain has at least one β-strand partially contained in both sheets [3]. In contrast, NTD and CTD are found in both ends of the sequence surrounding PDZ domains. NTD is associated with the structural role of the protein by its interaction with the PDZ domain [4], while CTD has a structural function related to homodimerization and oligomerization during the interaction with the syndecan-4 complex [18]. Finally, PDZ domains are responsible for the interaction by binding with the C-terminal of multi-protein complex [3,4]. The major binding activity is attributed to PDZ-2, including class 1 PDZ binding proteins (neurofascin, neuroglian-180, and pro-TGF-α) that contain threonine or serine residues in the C-terminal domain. Additionally, PDZ-2 interacts with class II PDZ binding proteins (syndecans, neurexins I-III, class B Ephrins, and EphA7) that contain aromatic or hydrophobic residues in the C-terminal domain [3,4,19]. On the other hand, the PDZ-1 domain binds to class I and III peptides such as CD63 [20], neurexin (NRXN) [4], and IL-5Ra [21]. In addition, the NTD domain can interact with ALIX [22], and finally, the CTD interacts with Rheb [23] and CD63 [20] (Figure 1).

### 2.1. Biological Role of Syntenin-1

**Figure 2:**
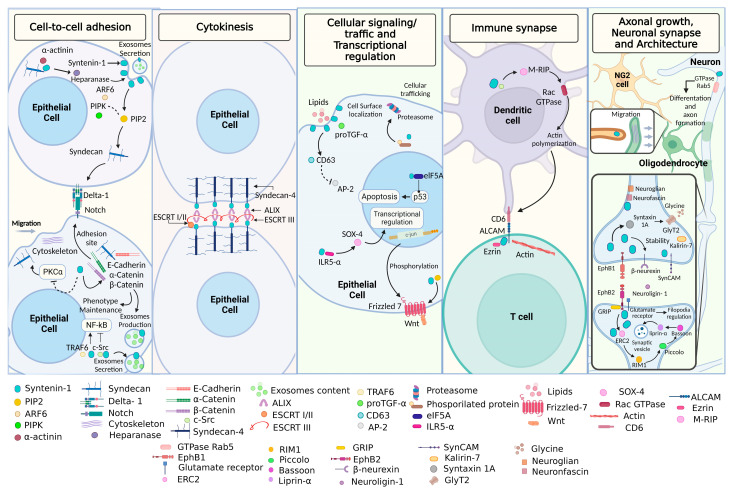
Biological functions of syntenin-1. The expression of syntenin-1 allows cell-to-cell adhesion by the interaction with molecules such as Delta-1, E-cadherin, TRAF6, and PIP2. Syntenin-1 participates in the signaling and cellular traffic, along with proTGF-α. For transcriptional regulation, syntenin-1 interacts with Frizzled-7, PIP2, IL5RA, and Wnt, whereas, for cytokinesis, interacts with molecules such as ALIX and syndecans. During the immune synapse, syntenin-1 relates with ALCAM and ezrin; finally, in the neuronal synapse, syntenin-1 interacts with neurexin, neurofascin, GlyT2, glutamate receptors, and beta Ephrin. Image created in BioRender (www.biorender.com, accessed on 14 February 2023).

Syntenin-1 is involved in several biological functions, such as cell-to-cell adhesion, cellular signaling and trafficking, regulation of transcription, immunological synapse, axonal growth, neuronal synapse, and architecture, due to its ability to interact with several molecules, including proteins, glycoproteins, and lipids (Figure 2).

#### 2.1.1. Cell-to-Cell Adhesion and Cytokinesis

Syntenin-1 interacts with phosphatidylinositol 4–5 bisphosphate (PIP2) recruited by PIPK and ARF6. During its localization in the plasmatic membrane, syntenin-1 modulates the recycling of syndecans, which gives place to the superficial availability of signaling molecules and cell adhesion [24,25,26]. Cytoskeleton organization and cell migration could be affected by the oligomerization of syndecan-4 mediated by syntenin-1 by inhibiting the activation of PKC-alpha (PKC-α). Additionally, syntenin-1 can interact with the sequence FYA in the C-terminal of syndecan-1 and co-localize with E-cadherin, β-catenin, and α-catenin in the filopodia (adhesion sites), microfilaments and nuclei, for the maintenance of the epithelial phenotype by affecting the organization of actin in the cytoskeleton and the production of exosomes [1,6,18,24]. Exosome production is regulated by TSPAN6, which recruits syntenin-1 [27], and the complex syntenin-1/syndecan/ALIX, whose secretion is positively regulated by heparanase. Mainly, its processing depends on α-actinin, syndecan, and syntenin-1 [22,28,29]. The complex syntenin-1/syndecan-4/ALIX is necessary to enrich the ESCRT-III machinery, and its coupling to the membrane contributes to cytokinesis [30]. Syntenin-1 interacts with c-Src for exosome secretion with pro-migratory activity in endothelial cells, which has been related to the modulation of ARF6 [31]. This interaction is essential for activating NF-κB triggered by IL-1R and TLR4 and mediated by TRAF6. Therefore, syntenin-1 is a negative regulator of NF-κB activation [32]. Finally, syntenin-1 could be related to cell adhesion and differentiation by forming a complex with Delta-1 and by participating in the signaling regulation of Notch [33].

#### 2.1.2. Cellular Signaling/Trafficking and Transcriptional Regulation

Syntenin-1 facilitates the recruitment of ubiquitylated proteins to their corresponding protein complexes due to its binding with ubiquitin via LYPSL regulated by the phosphorylation of serine by Ulk1; this ubiquitination is related to the proteolysis and intermembrane traffic [34,35]. During the traffic of membrane lipids, syntenin-1 interacts with tetraspanin as proTGF-alpha for cell surface localization [36,37] or CD63 for internalization inhibition mediated by AP-2 and affecting the endocytosis [20]. The interaction of syntenin-1 with PIP2 and Frizzled-7 allows the modulation of the Wnt signaling pathway and the phosphorylation of c-jun. In addition, the complex syntenin-1/IL5RA could activate SOX-4; in both cases, the transcriptional regulation is promoted [38,39,40,41,42]. On the other hand, although the overexpression of elF5A regulates the positive expression of p53, its binding to syntenin-1 disrupts this regulation and promotes apoptosis [43].

#### 2.1.3. Immune Synapse

Syntenin-1 interferes in the interaction between dendritic cells and T cells through the polymerization of actin mediated by Rac GTPase, which is also regulated by M-RIP, whose processing is possible due to syntenin-1 phosphorylation by c-Src [44]. Additionally, the complex ALCAM-syntenin-1-Ezrin that interacts with CD6 allows the coupling of actin during the immunological synapse [45,46].

#### 2.1.4. Axonal Growth and Neuronal Synapse and Architecture

Syntenin-1 could interact with several molecules involved in axonal growth, architecture, and neuronal synapse. A higher expression of syntenin-1 has been reported in the brain tissue of rats [4], and it is co-localized with ERC1b in the brain; ERC2 in the olfactory bulb, cortex, and medial habenula; and with both in the hippocampus. The binding gives this interaction with the PDZ domains and the multimerization of syntenin-1 (presynaptic syntenin-1); however, this protein could be distributed to axons and dendrites by itself. This process starts in the cytoskeletal matrix located in the presynaptic zones (CAZ), in which several proteins are concentrated, such as Aczonin/Piccolo, Bassoon, RIM, Munc13, ERC, and Liprin-alpha. The interaction of syntenin-1 and ERC is possible due to the recruitment of syntenin-1 by EphB1 and EphB2, which are responsible for presynaptic development. EphB interacts with glutamate receptors and regulates the dendritic filopodia mediated by syntenin-1, which interacts with ERC2 and the CTD domain of RIM-1, Piccolo, Bassoon, and Liprin-alpha, allowing the binding of RIM1 to the synaptic vesicle that contains Rab3A [19,47,48,49,50]. The interaction of syntenin-1 with the Rab5 GTPase modulated by SynGAP, expressed in the axons and linked directly to Unc51, is responsible for neuronal differentiation and axon formation [51].

In addition, syntenin-1 can bind to Syntaxin 1A and modulate the traffic and the presynaptic localization of GlyT2 for the reuptake of glycine in the extracellular space [52]. It interacts and activates the function of beta-neurexin (receptor for neuroligin-1), which is involved in the formation of synapses and contacts of the exon by the joint signaling with other transsynaptic factors such as Wnt, cadherins, PICK1, and APP. In the same way, syntenin-1 can bind to neurofascin, and neuroglian, belonging to the family of L1 transmembrane proteins, responsible for cell adhesion, growth, and maintenance of the axon [4,47,53,54,55,56]. Syntenin-1 interacts with SynCAM, related to accelerating maturation and improving synapse stability in the dendritic spine. Synapse stability has also been associated with the interaction of syntenin-1 with GlyT2 [57,58,59,60,61] and Karilin-7, which are redirected to the postsynaptic density fraction (PSD) for dendritic morphogenesis regulation through Rac1 signaling with neurabin, spinophilin, and AF-6 to the actin cytoskeleton. Finally, syntenin-1 connects NG2 with cytoskeletal machinery for oligodendrocyte migration and binds to glutamate receptors, (KAR) GluR52b, GluT52c, and GluR6, forming protein complex along with PSD5, GRIP, and PICK1; the last two interact to keep the synaptic function by redirecting to PKC for the phosphorylation of the KAR [62,63,64,65].

### 2.2. Syntenin-1 and Cancer Development

Syntenin-1 participates in normal biological functions; however, its overexpression has been associated with tumor development in lung cancer (small and non-small), colon, pancreas, prostate, breast, and glioblastoma. Syntenin-1 is also implicated in cell migration, metastasis, tumor growth, and angiogenesis. The role of syntenin-1 in tumor development has been associated with its interaction with syndecan, proTGF-alpha, and beta Ephrins, which could be inhibited by r-PTP, a tumor suppressor [66]. Syntenin-1 suppression promotes lymphocyte T activation via STAT3/IL-1β, associating its suppression with the activation of the immune system against cancer [13]. The expression of syntenin-1 could be inhibited by the phosphorylation of ERK in the pathway MAPK [8]. The interaction of syntenin-1 with heparanase mediated by syndecan-1 and α-actinin has been associated with metastasis and its capacity to release exosomes [29]. Syntenin-1 also participates in exosomal communication between tumoral cells. The release of extracellular vesicles mediated by this protein and syndecans could promote an increase of the onco-miRNA: miR-181a, miR-425-5p, and miR-494-3p, which promotes cell growth, migration, and angiogenesis in lung cancer [10,67]. Syntenin-1 is located in the extracellular vesicle with CD-81, CD63, VAMP3, GTPases, and integrin [68].

**Figure 3:**
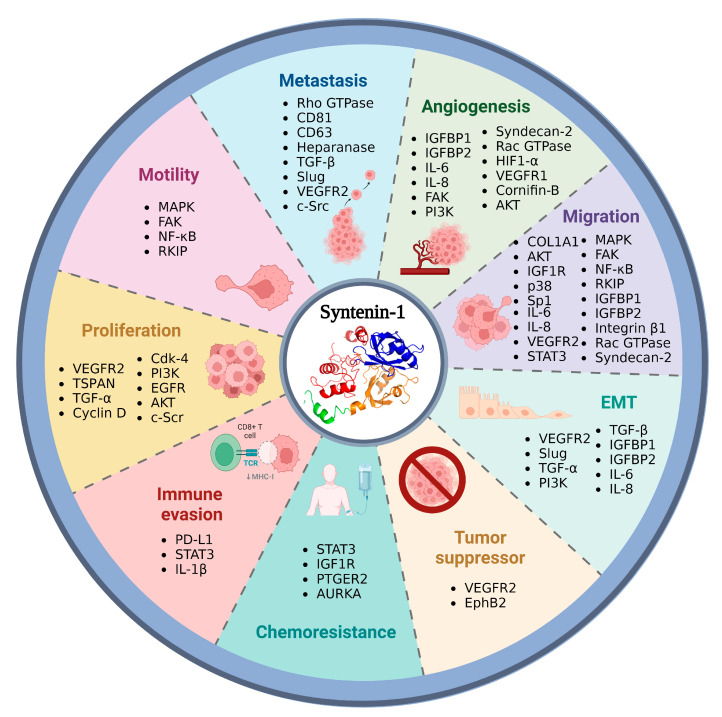
Overexpression of syntenin-1 and cancer development. The overexpression of syntenin-1 is related to angiogenesis (IGFBP1, IGFBP2, Rac GTPase, PI3K, Syndecan-2, HIF1-α, and SPRR1B), metastasis (TGF-beta, Rho GTPase, Slug, c-Src, Heparanase), migration (COL-1, AKT, MAPK, p38, SP1, and Syndecan-2), EMT (TGF-beta, VEGFR, IL6, and IL8), chemoresistance (STAT3, IGF-1R, PTGER2, and AURKA), proliferation (Cdk-4, TSPAN, Cyclin D, TGF-alfa, and s-Src), and motility (MAPK, FAK, NF-kB, and RKIP), also shows a tumor suppressor role by its interaction with VEGFR2 and EphB2. Image created in BioRender (www.biorender.com, accessed on 14 February 2023).

Syntenin-1 participates in cancer progression, particularly in motility and cell invasion, by its association with FAK and MAPK in signaling pathways that activate NF-κB. It could be negatively regulated by its interaction with syntenin-1 and RKIP [69,70,71]. Additionally, syntenin-1 regulates the activation of Smad, induced by TGF-beta, and cell invasion and metastasis [72]. This protein forms complexes with VEGFR2 and EphB2, suppressing the VEGFR2-depending angiogenesis in endothelial cells [73]. In colorectal, breast, and melanoma, syntenin-1 favors migration and tumoral growth by controlling integrin β1 and levels of Cdk4 and cyclin D2 [74] (Figure 3).

#### 2.2.1. Colorectal, Pancreatic, Prostate, and Gastric Cancer

In colorectal cancer, syntenin-1 influences cell migration and chemoresistance by regulating PTGER2 [11], whereas its interaction with TSPAN6 and TGF-alpha in extracellular vesicles promotes tumor growth [75]. Syntenin-1 promotes proliferation, cell migration, invasion, and EMT in pancreatic cancer by interacting with PI3K/AKT. Additionally, it promotes invasion, cell migration, and angiogenesis in prostate cancer by interacting with IGFBP1 and positive regulation with IGFBP2, IL-6, IL-8, and VEGFA. In prostate cancer, syntenin-1 activates STAT3 by its interaction with IGF-1R; thus, it promotes invasion and cell migration, which is also regulated by Rac by the interaction with syntenin-1 and syndecan-2 [9]. The activation of STAT-3 has also been related to chemoresistance regulation and the stemness of prostate tumoral cells [76]. Furthermore, the phosphorylation of STAT3 by syntenin-1 promotes growth and metastasis in gastric cancer [77,78]. In this type of cancer, the overexpression of syntenin-1 can induce cell proliferation by the PI3K/AKT pathway [79].

#### 2.2.2. Lung, Melanoma, and Breast Cancer

The overexpression of syntenin-1 in lung cancer has been associated with VEGF, promoting tumor growth and metastasis [80], and in complex with Slug, it can regulate EMT and metastasis [14]. In melanoma, syntenin-1 activates c-Src, and its overexpression is related to metastasis and cell migration. It also promotes angiogenesis by affecting the expression of pro-angiogenic genes by interacting with the extracellular matrix and activating c-Src and FAK, which allows the phosphorylation of Akt to produce HIF1-α [69,81,82,83,84,85]. Syntenin-1 interacts with TGF-β, enables the activation of Smad, and participates in the EMT in lung cancer cells activated by Ras [86]; the invasion can be promoted by activating p38, MAPK, AKT, FAK, and SP1 [87]. The interaction with TGF-beta has also been associated with the modulation of the GTPase RhoA, Cdc42, and TGFB1 in the EMT of breast cancer [88]. In breast cancer, the interaction of syntenin-1 with ESCRT promotes the activation of pro-tumoral fibroblast [89]. Additionally, it can regulate the expression of pEGFR and PBCL2 to inhibit autophagy markers [12], and its interaction with TGF-β can give place to proliferation in EMT [86]. The activation of AKT mediated by syntenin-1 facilitates the function of COL-1 [90]. It activates the integrin β1 and ERK1/2 in breast cancer, thus promoting cell migration and invasion [91] by participating in the signaling Ras, Rho, PI3K, and MAPK. In addition, it induces immune response evasion by the positive regulation of PD-L1 in triple-negative breast cancer [92]. In addition, the role of syntenin-1 in breast cancer during the growth, metastasis, and proliferation could be deleted by miR-216b and miRNA-139-3p [93].

#### 2.2.3. Glioblastoma, Hepatocarcinoma, HNSCC, and Urothelial Cell Carcinoma

The viability of glioblastoma cells [94] and the regulation of stemness genes such as Nanog, Oct4, and Sox2 related to the activation of STAT3 and cell migration through the signaling of FAK-JNK and FAK-AKT have been associated with syntenin-1 [95,96]. In other cancers, such as Neurofibromatosis type II, syntenin-1 interacts with Merlin [3], and in hepatocarcinoma, it increases the proliferation and invasion by affecting the levels of EGFR and MMP-2 and activating NFκB [97,98]. In uveal melanoma, syntenin-1 expression has been related to the invasion mediated by the hepatocytes’ growth factor [99]. Additionally, it regulates the differentiation and angiogenesis in head and neck squamous cell carcinoma (HNSCC) by interacting with SPRR1B and VEGFR1 [100], and the phosphorylation of syntenin-1 mediated by AURKA promotes the progression of the HNSCC [101] and allows the modulation of the stemness and chemoresistance [102]. In addition, in urothelial cell carcinoma, syntenin-1 regulates the proliferation and invasion by modeling the signaling pathway of EGFR, AKT, PI3K, and c-Src, as well as the progression of EMT by the alteration in the expression of β-catenin, E-cadherin, vimentin, Claudin-1, ZO-1, and TCF4 [103]. In pheochromocytoma, syntenin-1 has been related to the complex COL3A1, COL5A2, and SERPINE1 [104]. Finally, syntenin-1 can modulate the internalization of the oncogenic human papillomavirus (HPV) in complex with CD63 [105].

## 3. Syntenin-1 as a Therapeutic Target in Cancer

### 3.1. Lung Cancer

**Figure 4:**
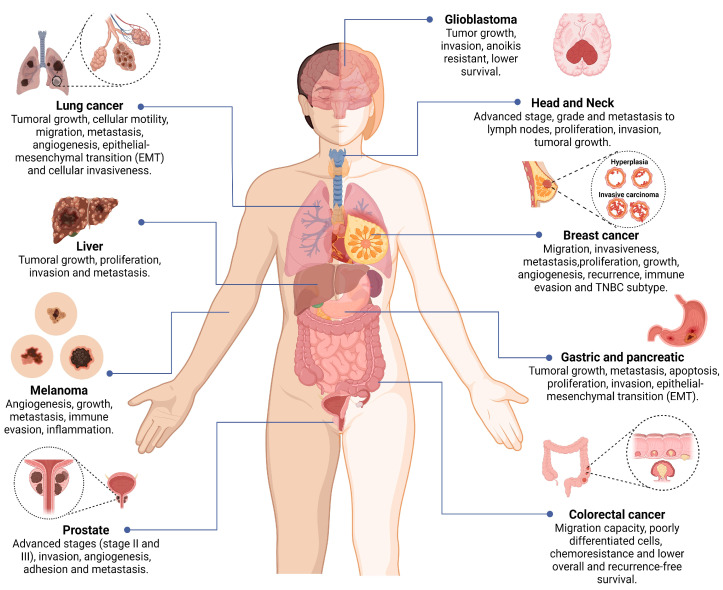
Role of syntenin-1 in the development of different types of cancer. The overexpression of syntenin-1 has been associated with the progression of several cancers by promoting tumor growth, migration, invasion, immune response evasion, proliferation, angiogenesis, and metastasis. Additionally, it has been associated with advanced stages, overall survival, and recurrence. Tumors with an overexpression of syntenin-1 include melanoma, prostate, colorectal, and breast cancer, followed by glioblastoma, gastric, pancreatic, liver, and HNSCC. Image created in BioRender (www.biorender.com, accessed on 14 February 2023).

In tissue samples from patients with small cell lung cancer, positive expression of syntenin-1 correlated with extensive and advanced disease (54.2% of SCLC). This suggests that syntenin-1 could be related to the development of neuroendocrine tumors, which can be attributed to the production of metalloproteinase that improves cell motility, invasion, and tumoral growth [87]. In serum samples and tissues with lung cancer (n = 191; SCC: Squamous Cell Carcinoma, LCLC: Large Cell Lung Carcinoma, SCLC: Small Cell Lung Cancer), the overexpression of syntenin-1 was correlated with the size (*p* = 0.002), stage (*p* = 0.020), long-distance metastasis (*p* = 0.033), and angiogenesis of the tumor. A positive correlation between serum levels of syntenin-1 and the vascular endothelial growth factor (VEGF; r = 0.49 and *p* ≤ 0.001); also, the overall and disease-free survival were shorter in patients with high levels of this protein [80]. A knockdown of syntenin-1 using cell lines derived from lung cancer (NCI-H1299 and NCI-H226) shows that the axis Ras/Syntenin-1 stimulated the release of extracellular vesicles charged with onco-miRNA to promote the tumor growth, cell migration, metastasis, and angiogenesis. In the same study, the treatment of other tumoral cell lines (A549, MDA-MB-231, and B16F10) with isolated exosomes reduced the abovementioned process. Interestingly, they concluded that extracellular vesicles mediated by syntenin-1 were crucial for the induction of cell migration, metastasis, and angiogenesis of tumor cells [10]. Kim et al., using siRNA targeting syntenin-1 in lung cancer-derived cell lines (CL1-5 and CL141), observed a decrease in the invasiveness and major cell-cell adhesion. These findings suggested that syntenin-1 could promote EMT and cellular invasiveness. Complementary, the overexpression of this protein in HEK293 cells increased invasion, whereas, during in vivo studies in a murine model, results were traduced in more metastatic nodules in lungs [14] (Figure 4).

### 3.2. Colorectal, Prostate Cancer, and Glioblastoma

In cell lines of colorectal carcinoma (SW480, SW620, and Caco2) treated with shRNA against syntenin-1, Iwamoto et al. observed a diminution in the migration capacity and chemoresistance to L-OHP (Oxaliplatin) in the three cell lines. The sphere formation assay revealed a diminution in the number of tumor stem cells in shRNA-treated cells. A complementary analysis in tissue samples from patients with colorectal carcinoma (n = 139) showed that the overexpression of syntenin-1 was associated with less differentiated histological stages (*p* = 0.001). In contrast, low expression was correlated with higher overall and recurrence-free survival (*p* ≤ 0.0001) [11]. In prostate cancer (PCa), overexpression of syntenin-1 was considerably higher in samples derived from patients with stages II and III (n = 64). To evaluate the potential relationship between syntenin-1 with the invasion capacity, this protein was overexpressed in the cell line RWPE-1 (non-tumor human prostate epithelial cell line). It promoted an increase in invasion capability. Additional studies were conducted in aggressive PCa-derived cell lines (PC-3, DU-145, and ARCaP-M) treated with shRNA against syntenin-1 to reduce cell invasion. The authors suggested that the PDZ1 domain mainly promotes invasion instead of PDZ2 in PCa cells (*p* = 0.0002) [80]. Later, authors designed a small-molecule inhibitor against the PDZ1 domain named PDZ1i that was tested in different cell lines of PCa (RWPE-1, DU-145, and ARCaP-M). The PDZ1i (50 µmol/L) was not toxic, altered the adhesion, and decreased invasion in cell lines with a positive to syntenin-1 expression. This was attributed to the inhibition of the physical interaction with IGF-1R and STAT3 activation, which affected the invasion and angiogenesis of PCa [15]. In multiform glioblastoma, syntenin-1 regulates and maintains anoikis-resistant glioma stem cells through protective autophagy. The loss of syntenin-1 deregulates the signaling pathways involved and promotes an excess in the levels of autophagy that change from protective to toxic [106]. Complementary assays using PDZ1i in glioblastoma in vivo models, PDZ1i resulted in smaller and less invasive tumors and enhanced survival. The authors concluded that PDZ1i could be a promise in treating advanced brain cancer [107] (Figure 4).

### 3.3. Breast Cancer

During the analysis of genes differentially expressed in breast cancer-derived cell lines (MDA-MB-231 and MCF7), the overexpression of syntenin-1 in the cell line MDA-MB-231 was correlated with the migratory potential, invasiveness, and metastasis, potentially associated with the PDZ2 domain. Results demonstrated that syntenin-1 could function as a metastasis-inducing gene [78]. In breast cancer tissue samples (n = 160), syntenin-1 was overexpressed mainly in negative tumors to the estrogen receptors (ER) (80.6%, *p* = 0.001). In vitro studies performed after the silencing of syntenin-1 showed attenuation in the growth rate of MDA-MB-231 cells, whereas, in nude mice, the tumor volume was significantly lower (90%, *p* ≤ 0.05) in shRNA-treated mice. These data suggested that syntenin-1 can activate alternative signaling pathways associated with proliferation when the estrogen signaling pathway is unavailable, as triple-negative subtypes, where the negative expression of receptors decreases the efficacy of most treatments [108,109].

Through in vivo and in vitro studies in breast cancer using shRNA against syntenin-1, Yang et al. established an association between cell migration, invasion, angiogenesis, growth, and metastasis with the expression levels of syntenin-1. In samples derived from patients (n = 239), a positive correlation between the expression of syntenin-1 and tumor size (*p* = 0.011), metastasis to lymph nodes (*p* = 0.001), and recurrence (*p* = 0.002) [92]. The pharmacological inhibition with PDZ1i in a murine model of breast cancer confirmed the reduction of metastatic nodules and the number of Myeloid-derived suppressor cells (MDSC), which was related to reduced metastasis and the systemic activation of T cells after the treatment [13]. Considering all the aforementioned, syntenin-1 has been proposed as a potential target for designing complementary therapies focused on blocking the metastatic process in breast cancer (Figure 4).

### 3.4. Melanoma

Concerning the participation of syntenin-1 in melanoma, studies in cell lines (C8161.9 and FM-516 SV) and immunodeficient animals showed that the protein was silencing (knockdown) or overexpressed. The knockdown inhibits angiogenesis and tumoral capacity of the cells C8161.9, which was traduced in the reduction of volume and weight of the tumors. While the overexpression of the protein in cells FM-516 SV showed opposite results. The evaluation of MDSC was significant in tissues with overexpression of syntenin-1 and suggested the potential role of the protein in promoting tumoral microenvironment alterations that leads to dysregulation of inflammation to support the tumor [81,82,83]. An overexpression of syntenin-1 was reported in primary uveal melanoma (n = 29) and was related to advanced stages (*p* = 0.009) and aggressive subtypes, whereas the silencing of syntenin-1 in cells 92.1 and Mel270 affected migration and invasion capacity. Additionally, phenotypes with overexpression of syntenin-1 were correlated with a higher risk of metastatic resistance, suggesting a potential role as a progression marker [99] (Figure 4).

### 3.5. Head and Neck Cancer

The expression of syntenin-1 in Head and Neck Squamous Cell Carcinoma (n = 81) was associated with the stage (*p* = 0.001), grade (*p* = 0.001), and lymph node metastasis (*p* = 0.0001). Using shRNA against syntenin-1, a diminution in the proliferation (*p* = 0.004), invasion (*p* = 0.01), and anchorage-independent growth (*p* = 0.02) was reversed after syntenin-1 re-expression due to the association of the PDZ1 domain of syntenin-1 with other molecules [101]. By comparing the proteome of cells UM1 (highly invasive) and UM2 (less invasive), the presence of membrane proteins and membrane-associated proteins as syntenin-1 was reported. Using siRNA against the protein and after several assays, an association of syntenin-1 with cellular migration, proliferation, and invasion of cell lines of HNSC was reported. In samples derived from patients, the expression of syntenin-1 was higher in HNSC tissue samples compared to healthy tissue. Additionally, the positive regulation of syntenin-1 was associated with metastasis to lymph nodes, survival rate, and recurrence [110]. Considering all the collected evidence, the clinical relevance of syntenin-1 in the biological process associated with cancer development and progression is undeniable and impulses the potential use as a therapeutic target in developing and improving targeted therapies (Figure 4).

## 4. Conclusions

Given the function of syntenin-1 as a scaffolding protein, it interacts with different cell types, which mediates essential functions such as traffic, cellular signaling, the biogenesis of exosomes, and transcriptional regulation to promote cell growth. In neurons, syntenin-1 is related to growth and axonal maintenance, as well as neuronal synapse. These functions are attributed to its capacity to mediate protein-protein interactions and stimulate cell adhesion. The PDZ domains are associated with the adequate function of the protein and its participation in the development of diseases such as cancer. The overexpression of syntenin-1 has been reported in melanoma, lung, prostate, and breast cancer, which promotes tumorigenesis by regulating cell migration, invasion, proliferation, angiogenesis, apoptosis, immune response evasion, and metastasis. It has been proposed the potential use of syntenin-1 in the development of novel strategies targeting specific domains of the protein for the inhibition of all biological functions associated with its overexpression. Among them, we can find shRNA, siRNA, and PDZ1i, which diminished the size and reduced invasion and metastasis. However, new strategies must be explored, such as antibodies targeting PDZ1 and PDZ2 domains for its administration as passive therapy or synthesizing recombinant proteins or peptides derived from syntenin-1 that can be used in the design of a novel vaccine for its administration as active therapy.

However, although exciting information has been described the potential role of syntenin-1 as a novel biomarker and potential therapeutic target. In vitro, in vivo, and the analysis of samples have some limitations. The expression of syntenin-1 in vitro using cell lines can oscillate according to the specific type of cancer, alterations in the cellular metabolism, and the experiment’s reproducibility. In vivo models have been performed in nude and Balb/C mice, in which the expression of syntenin-1 can fluctuate according to the type of cancer; also, the effectivity of the evaluated treatment cannot be reached in humans due to differences in the metabolism, the type/stage of cancer or physiological factors of the host. Finally, during the analysis of samples, some limitations associated with the clinical information of patients, such as previous treatments and the stage, were not included in the description. Nevertheless, there is a need to perform more studies focused on evaluating clinical samples derived from different stages of cancer to determine the potential role of syntenin-1 as an early prognostic biomarker of invasion and metastasis in all those cancers its overexpression has been reported. To overcome these limitations, further studies using in vitro and in vivo models need to be performed to corroborate the potential role of syntenin-1 as a prognostic biomarker and therapeutic target in cancer; interestingly, promising results are found in the bibliography.

## Data Availability

Data sharing is not applicable.
